# Relative contribution of reproductive attributes to the density-dependent effects on fruit-set

**DOI:** 10.1093/aobpla/ply019

**Published:** 2018-03-13

**Authors:** Vineet Kumar Singh, Chandan Barman, Divya Mohanty, Rajesh Tandon

**Affiliations:** Department of Botany, University of Delhi, New Delhi, India

**Keywords:** Inbreeding depression, myophily, partial self-compatibility, reproductive biology

## Abstract

Reproductive success of a plant species can be affected by the distribution pattern of its conspecifics in a small population. Besides the low mate availability, the dynamics of breeding system and pollination mechanism may also contribute to low fruit-set in such populations. We examined the relative contribution of these reproductive attributes on fruit-set across the contrasting distribution pattern (denser vs. sparser plots) in two isolated natural populations of a near-threatened tree species, *Anogeissus sericea* var. *nummularia*. Although flowers in the species are of generalist type, the narrow stigmatic surface appears to impose a requirement for a specialist pollinator. Pollination in the tree species is mediated only by the flies. The trees exhibit partial selfing and suffer from strong inbreeding depression at the early life-history stages of the selfed progeny. We recorded significant difference between the denser and sparser plots in terms of inflorescence visits per tree, and the number of trees covered in a bout by the pollinators. Moreover, tree density showed a strong positive correlation with fruit-set. Besides the requirement of having proximity among the conspecifics to facilitate pollinator movement, pollen quality also seemed to be a crucial attribute in the reproductive success of the tree species. It is inferred that the mating pattern and fecundity of plants in small and isolated populations are significantly influenced by the extent of sexual incompatibility and magnitude of their dependence on pollinators.

## Teaser

The reproductive success of biotically pollinated plant species usually declines with population density. However, the contribution of the prevailing breeding system and pollination mechanism in a particular demographic set-up is also crucial to determine the success. We examined the relative contribution of these factors in a threatened tree species *Anogeissus sericea* var. *nummularia*. The study shows that besides the breeding system of the species, the extent of pollinator dependence and its foraging behaviour significantly contributes to the net outcome of density-dependent effects.

## Introduction

Plant communities in arid and semi-arid zones are usually small or isolated and their reproductive success is constrained by low mating opportunities (Aguiar and Sala 1999). This is largely because the interaction dynamics of certain reproductive attributes become confined due to the sparser distribution pattern of the conspecifics. Among these attributes, pollination mechanism and breeding system of biotically pollinated plant species are of significance (Silander 1978; Kunin 1992, 1997; Ghazoul *et al.* 1998). In a density-dependent landscape, the extent of pollinator dependence and sexual compatibility play a crucial role in defining the net outcome of mating success. Knowledge of interaction between these reproductive attributes is vital and is considered a prerequisite in attempts to recover threatened plant species (Metcalfe and Kunin 2006; Ruane *et al.* 2014).

Density-dependent effects on mating pattern can be ascertained from the contributors of pollination success such as the amount of stigmatic pollen load, pollination efficiency and the foraging behaviour of pollinators (Schemske and Horvitz 1984; Bosch and Waser 1999; Mustajärvi *et al.* 2001; Bernhardt *et al.* 2008). Decline in patch size leads to discounting of pollen not only in terms of quantity and but also the quality (compatibility), which may proportionally influence the extent of fruit-set and seed-set (efficiency). Thus, in sparser landscapes there is likely to be a limited deposition of compatible pollen onto the stigma. Highly scattered floral resources for pollinators also can have a varied outcome depending on the extent of mutualism (specialist vs. generalist) (González-Varo *et al.* 2009), and the efficiency of foraging movements by the pollinators (Lehtilä and Strauss 1997; Somanathan and Borges 2000, 2001). In accordance with the optimal foraging theory (Pyke 1984), fruit-set may enhance in denser patches, as they confer mass floral display and greater amount of floral resources to the pollinators (Rathcke 1983; Cresswell and Osborne 2004; Castilla *et al.* 2016). Pollinators tend to visit patches having flowering individuals in proximity (Heinrich 1979; Pyke 1984; Chi and Molano-Flores 2014). In sparser patches, the pollinators generally have to cover greater number of flowers per plant to suffice their energy requirement than in the denser patches. Reproductive fitness would increase in denser patches, provided there is no competition for resource and/or pollinators among the conspecifics (Rathcke 1983). Outcomes of pollination efficiency within a density-dependent scenario can also be linked to the ability of pollinators to cover a certain number of plants. Pollinators that tend to cover fewer plants in their foraging bouts result in enhanced fruit-set than those that cover relatively larger area.

Depauperate populations of preferential outbreeding plants are known to experience higher inbreeding depression (ID) and reduced fitness of offspring (Barrett and Kohn 1991; Ellstrand and Elam 1993). These effects may be similar in ecological settings with variable density distribution of plants. However, this aspect has not been sufficiently investigated.

Although the impact of density of plants on fecundity has been frequently investigated (Donaldson *et al.* 2002; Pan *et al.* 2012), there is paucity of information on the relative contribution of reproductive features to the net outcome of fruit-set in such environments. The enormous variability in sexual and breeding system, pollination mechanism and combinations thereof among the flowering plants may generate different outcomes in uneven density-dependent landscapes. In the present work, we demonstrate the sensitivity of these attributes to the distribution pattern of conspecifics within a population in a near-threatened tree species, *Anogeissus sericea* var. *nummularia*. We first investigated the floral biology in relation to pollination mechanism and breeding system of the species, and then compared the contribution of the pollinator and its behaviour in net fruit-set in contrasting patches of plant densities. We have shown that the effect of plant density on fruit-set in small populations is significantly contributed by the foraging behaviour of the pollinator and sexual compatibility in the species.

## Methods

*Anogeissus sericea* var. *nummularia* is a semi-deciduous and a near-threatened tree species (Meena 2013). The tree is confined to some isolated populations in the arid and semi-arid regions of Rajasthan. The trees flower for a period of 6–8 weeks in ‘cornucopia’ type pattern (Gentry 1974).

### Study design

The study was conducted for two consecutive seasons in two natural populations located at two different sites: Bharthari, Sariska (ASNA) (N 27°23.935/E 76°24.179) at Alwar (~125 trees) and Kayalana (ASNJ) (N 26°18.199/E 72°58.580) in Jodhpur (~70 trees), Rajasthan. The other perennial plants at the sites included three species each of trees (*Anogeissus pendula*, *Senegalia senegal* and *Prosopis juliflora*), and shrubs (*Justicia adathoda*, *Zizyphus nummularia*, *Calatropis procera*). Among these, *J. adathoda* was the only co-flowering species at the site, which was visited by *Apis dorsata* and *Xylocopa.*

The distribution pattern of trees within the populations at both the sites was greater at the centre than the periphery. We marked two contrasting plots (~1.25 ha) at each of the two study sites, one plot with denser distribution of trees (3 to 6 trees per 100 m^2^) and the other with sparser (1 or 2 trees per 100 m^2^). The distribution pattern (trees per 100 m^2^) did not differ among the two populations (*t* = 0.874, df = 1,18; independent sample *t*-test). The distance between the two plots at each site was nearly 100 m and the plots had the same floristic composition.

### Floral biology

The average number of flowers borne in an inflorescence (*n* = 100) was recorded from a set of randomly marked branches (*n* = 10) on 10 trees. The time of anthesis and anther dehiscence was noted by monitoring the randomly tagged inflorescence (*n* = 50). Pollen production in a flower (*n* = 10 flowers) was determined using a haemocytometer (Kearns and Inouye 1993). Ovule production in a flower was averaged from 50 inflorescences. The stigma receptivity was determined by localizing peroxidases (Dafni *et al.* 2005). The viability of the pollen was ascertained by the fluorochromatic reaction (FCR) test (Heslop-Harrison and Heslop-Harrison 1970).

### Breeding system

The breeding system was established through controlled pollinations performed during the two seasons. As the flowers were very small, the entire inflorescence was bagged after removing the older flowers. Four types of pollination treatments were performed on these marked inflorescences: (i) spontaneous autogamy—inflorescences were bagged 1 day before anthesis without emasculation; (ii) facilitated autogamy—the emasculated flowers of inflorescence were pollinated with pollen from the inflorescence of the same tree; (iii) xenogamy—the emasculated inflorescence were pollinated with pollen grains sourced from a different tree; and (iv) apomixis—inflorescences were emasculated and bagged without pollination. A set of randomly tagged open-pollinated inflorescences were considered as control. Fruit-set and seed-set from all the treatments was monitored and recorded as percentage. To determine the mean pollen load on the stigmatic surface, open-pollinated inflorescences (*n* = 50 flowers, each population; 48 h after anthesis) were randomly collected and the amount of pollen was counted under an epifluorescence microscope, by staining the stigmatic surface with auramine O (Tandon *et al.* 2001). The index of self-incompatibility (ISI) was expressed as the ratio of percentage fruit-set from manual self-pollination to percentage fruits formed through manual cross-pollination (Zapata and Arroyo 1978).

### ID and seed germination

Inbreeding depression manifested at fruit-set/seed-set, seed germination and seedling stages was computed by using the equation δ_t_ = 1 − (*w*_s_/*w*_o_), where *w*_s_ and *w*_o_ referred to fitness of selfed and outcrossed offspring, respectively (Charlesworth and Charlesworth 1987). Seed germinability and survival were ascertained from five replicates of 15 seeds each, obtained from the two treatments. Seeds were scarified using sand paper (Flint paper-100) and dark-incubated in petri dishes lined with moist germination paper. Germinated seedlings (with radicle emerged) were transferred to pots (15 × 15 cm) filled with soilrite and watered regularly. Percentage seedling survival was recorded 1 month after germination. The seed weight (mg) was compared using two-way ANOVA with two populations and two flowering seasons as a fixed factor (*n* = mean weight averaged in each tree for 20 trees).

### Pollination ecology

Pollinator types and their behaviour were recorded through direct observations made during the peak time (mid-November to mid-December) of blooming (*n* = 10 trees; 15 days). Binoculars and SLR camera (Canon 600D) were used to record the observations. Floral visits were recorded during 30 min of observations at different hours of the day (0600–1800 h, GMT). During each interval, the following features (variables) were recorded: (i) visiting species and a legitimate or illegitimate floral visit; (ii) total number of inflorescence visited per tree; and (iii) inflorescence-handling time. To estimate the amount of pollen grains carried, each type of forager was captured using a fine net and collected in a vial having cotton soaked with minimal amount of ethyl acetate. Pollen grains were then removed from insect body in a 2 % sucrose solution by using fine camel hair brush and counted under a microscope (Dafni *et al.* 2005).

### Effect of tree density on pollination and natural fruit-set

The effect of tree density on pollinator behaviour and fruit-set was studied in the two plots of each population. The observations were recorded on both the plots during the peak flowering period, at an interval of 15 min each for seven consecutive days. The data were recorded simultaneously in both the plots by two different groups of two people each. We used the same pair of plots for two consecutive years to study the foraging behaviour of the pollinators (flies) in terms of (i) the mean number of inflorescence visited by flies per tree in a foraging bout, and (ii) the average number of trees visited per bout in a plot. The average number of trees covered in a bout was recorded by tracking the flies in the plot till they confined their foraging on a tree for a period up to 60 min or until it settled anywhere but the flowers. The observations on pollinator movement among the trees were recorded during the peak phase of flowering. The difference in pollinator behaviour in contrasting plots at each population was compared using three-way ANOVA. Where the two plots and the two populations were considered fixed factors and inflorescence visited per tree and trees visited per bout were individually considered as dependent variables.

For ascertaining the effect of density on fruit-set, trees (*n* = 15) were randomly marked in each plot at both study sites and on each of the marked tree we monitored 50 randomly tagged inflorescences until fruit maturation.

The effect of pollinator behaviour on natural fruit-set in relation to density of trees among the two plots was compared at both the sites using two-way ANOVA, where the two contrasting plots and the two populations were considered as fixed factors, and percentage fruit-set per tree as dependent variable. To understand the pattern of fruit-set in density-dependent set-up, the percent fruit-set was also correlated with the tree density per 100 m^2^. This was performed by randomly marking five quadrats each (10 × 10 m^2^) along the line transect within the denser and sparser plot at both populations and comparing the mean percentage fruit-set with the number of trees per 100 m^2^.

All data were tested for their normal distribution with Shapiro–Wilk test and compared by employing suitable tests using SPSS v. 22 (IBM® SPSS® Amos™ 22; IBM Corp. ver. 2013). *Post hoc* Tukey’s HSD test was used to ascertain homogeneity when variables were more than two. The percentage data were arcsine root-square transformed to achieve homoscedasticity (Sokal and Rohlf 2012) before performing the analyses.

## Results

### Floral biology

Flowering was a synchronized event, as nearly 75 % of the trees at each site simultaneously achieved the peak of flowering. Flower production neither differed significantly (*F*_1, 36_ = 2.69, *P* = 0.11, two-way ANOVA) between the denser (31.75 ± 0.6) and sparser plot (30.75 ± 0.87), nor (*F*_1, 36_ = 1.93, *P* = 0.174, two-way ANOVA) among the two populations, i.e. ASNA (31.6 ± 0.56) and ASNJ (30.55 ± 0.9). Similarly, there was no difference (*F*_1, 76_ = 0.20, *P* = 0.88, two-way ANOVA) in the mean inflorescence production per tree (19.65 ± 0.78 inflorescence per flowering branch) between the two types of plots.

**Figure 1.**
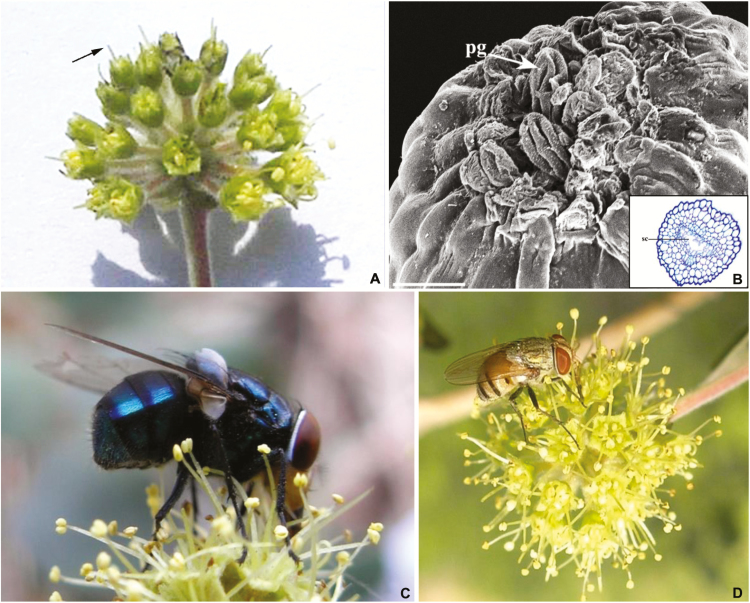
Floral features and pollinators of *A. sericea* var. *nummularia*. (A) An inflorescence at protogynous stage of flowers. Note that all the flowers are at the same stage in the inflorescence (arrow). (B) Scanning electron micrograph of a pollinated stigma. The pollen grains (pg) are deposited in the shallow cavity of the stigma (bar = 15 µm). Inset: transverse section across the base of stigmatic zone depicting the continuity with the stylar canal (sc). (C, D) *Chrysomya megacephalla* and *Musca* sp. foraging the flowers.

In general, anthesis began by 0430 h and most of the flowers (~70 %) in an inflorescence were opened by 0830 h. The freshly opened flowers are small (length = 7.11 ± 0.16 mm, *n* = 100) and grouped into terminal glomerulus (diameter = 12.35 ± 1.01 mm; height = 11.09 ± 0.83 mm; *n* = 100). The flowers are yellowish green and emit mild fetid smell when grouped together. Floral nectar was produced in trace amounts. On an average, a flower produced 11.4 × 10^4^ ± 0.31 pollen grains. Viability of fresh pollen was 88.43 ± 2.39 % in ASNA and 91.13 ± 1.85 % at ASNJ (*n* = 20 flowers). Flowers exhibited protogyny (Fig. 1A), as the stigma became receptive a day prior to anthesis while the anthers dehisced ~2 h after anthesis. The stigma is represented by a highly inconspicuous and shallow apical depression (Fig. 1B); only the inner lining of the stigma is receptive. The ovary contained two pendulous and anatropous ovules.

### Breeding system

Controlled pollinations established the occurrence of autogamy and absence of apomixis in the species (Table 1). However, xenogamy resulted in significantly greater yield (75–80 %) than the other treatments. Also, manual deposition of pollen grains (self or cross) gave greater fruit-set (55.25 %) than open-pollinations (24.98 %). This difference was significant (*t*-test, *t* = 7.328, df = 2, *P* = 0.018) and maintained at both the populations during the two seasons of study. Self-pollination resulted in 25.73 % seed-set and cross-pollination in 73.74 %. The ISI in the two populations averaged for 2 years was 0.4 (ASNA) and 0.42 (ASNJ).

**Table 1.** Outcome of the experimental pollinations during two seasons at the sites.

Pollination treatment	2012		2013	
	No. of treated inflorescence (flowers)	Fruit-set (%)	No. of treated inflorescence (flowers)	Fruit-set (%)
ASNA				
Spontaneous autogamy	50 (1824)	18.47	50 (1856)	20.2
Autogamy	50 (520)	35.58	50 (531)	29.38
Xenogamy	50 (490)	77.14	50 (464)	83.80
Apomixis	25 (85)	–	25 (124)	–
Open-pollination	50 (1805)	29.98	50 (1875)	27.89
ASNJ				
Spontaneous autogamy	30 (1165)	19.91	50 (1911)	18.27
Autogamy	30 (312)	36.22	50 (525)	32.11
Xenogamy	30 (300)	84	50 (500)	80.20
Apomixis	25 (100)	–	25 (100)	–
Open-pollination	30 (1141)	30.59	50 (1938)	28.99

### Inbreeding depression

Seed weight (*n* = 100) of the cross-progeny was 17.18 ± 0.3 mg while it was 8.48 ± 0.13 mg in the selfed ones. The seed weight of the inbred progenies was significantly lower (*F*_1, 76_ = 1698.86, *P* = 0.001, two-way ANOVA). Moreover, the percentage germination response of the seeds obtained through selfing (16.01 % ± 4.52) was significantly lower (F_1, 16_ = 41.051; *P* = 0.001; two populations five replicates of 15 seeds) than that from xenogamy (45.33 % ± 3.89). The percent seedling survival (up to 30 days) of cross-pollinated progeny at ASNA was 17.5 % and at ASNJ it was 26.67 % while that of the manually self-pollinated seeds was 6 % and 6.6 % at ASNA and ASNJ, respectively. The values of ID at different life stages were represented in Table 2. At all the mentioned stages values of inbreeding are invariably >0.5 (Table 2).

**Table 2.** Inbreeding depression (δ) manifested at different phases of plant development.

S. no	Development phase	δ ASNA	δ ASNJ	*N*
1.	Fruit-set	0.59	0.61	1051 (autogamy), 954 (xenogamy) at ASNA; and 837 (autogamy), 800 (xenogamy) at ASNJ
2.	Seed-set	0.61	0.63	1051 (autogamy), 954 (xenogamy) at ASNA; and 837 (autogamy), 800 (xenogamy) at ASNJ
3.	Seed weight	0.59	0.58	100 seeds each
4.	Seed germination	0.58	0.73	*n* = 80 (crossed), 50 (self) at ASNA and 75(crossed), 50 (self) at ASNJ
6.	Seedling survival	0.65	0.75	*n* = 80 (crossed), 50 (self) at ASNA and 75(crossed), 50 (self) at ASNJ

### Stigmatic pollen load

The average amount of stigmatic pollen load in the open-pollinated flowers was 8.74 ± 0.75 (*n* = 55) and 10.24 ± 0.59 (*n* = 50) in denser plot, and 5.82 ± 0.15 (*n* = 50) and 6.24 ± 0.74 (*n* = 50) in sparser one at ASNJ and ASNA, respectively. The mean amount of the stigmatic load was significantly greater (*H* = 154.02, *P* = 0.0001, Kruskal–Wallis) than the ovule number, i.e. 2 (*n* = 50, each population). Pollen load on the open-pollinated stigma was also greater than on the flowers bagged to ascertain spontaneous autogamy (1.58 ± 0.69, *n* = 50) (Mann–Whitney *U* = 789.500, *F*_1, 49_ = 2985.500, *P* = 0.001).

### Floral visitors

Flowers of *A. sericea* were visited by three species of fly (Table 3). Among these, *Chrysomya megacephala* was the most abundant and frequent visitor (Fig. 1C). They first arrived on flowers by 0800 h with increased frequency between 1030 and 1130 h. *Lucilia sericata* and *Musca* sp. were the other flies that visited the flowers (Fig. 1D). The three flies exhibited very slow and sluggish foraging behaviour and the inflorescence-handling time by flies was 2–6 min (Table 3). The pollen grains were mainly deposited sternotribically on the abdomen and setae of the tibia and tarsal region of the flies. Among the three flies, *C. megacephala* was most effective in carrying an appreciable amount of pollen load (Table 3).

**Table 3.** Pollinators and their behaviour on the trees of *A. sericea* var. *nummularia* at the two sites.

Pollinator (site)	Inflorescence-handling time (min)	Foraging frequency (*n* = 50)	Pollen load
(ASNA)			
*Chrysomya megacephala*	5.3 ± 0.19 (*n* = 30)	71.9 %	412.68 ± 45.9 (*n* = 20)
*Lucilia sericata*	2.7 ± 0.2 (*n* = 30)	17.35 %	290.78 ± 81.45 (*n* = 12)
*Musca* sp.	3.1 ± 0.2 (*n* = 30)	10.75 %	278.7 ± 31.15 (*n* = 15)
(ASNJ)			
*Chrysomya megacephala*	4.15 ± 0.25 (*n* = 50)	77.9 %	364.18 ± 65.57 (*n* = 15)
*Lucilia sericata*	2.15 ± 0.31 (*n* = 50)	9.45 %	187.89 ± 63.11 (*n* = 10)
*Musca* sp.	2.12 ± 0.55 (*n* = 30)	12.65 %	258.1 ± 24.11 (*n* = 10)

### Effect of tree density on natural fruit-set

**Figure 2.**
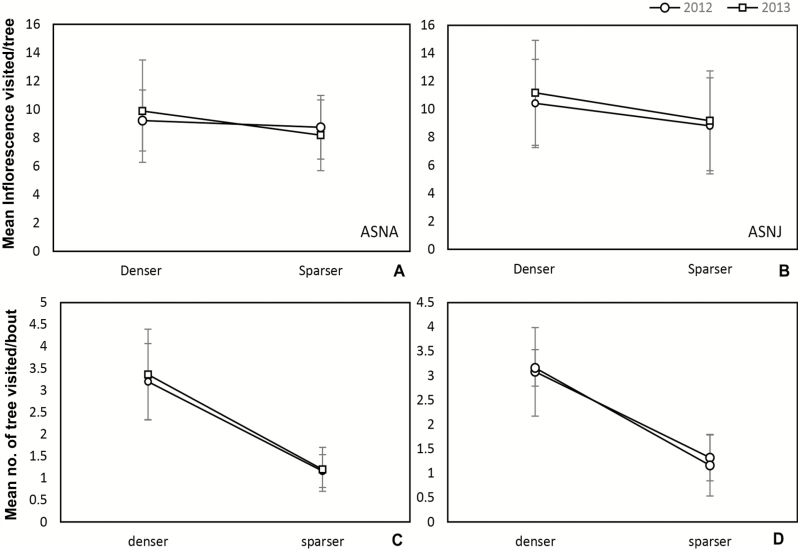
Effect of tree density on pollinator’s behaviour. Density significantly influenced the pollinator behaviour in terms of inflorescence (glomeruli) visited per tree and trees visited per bout by the pollinators. (A) and (B) show mean number of glomeruli visited by flies per tree; (C) and (D) represent mean number of trees visited per bout by the pollinators in sparser and denser plot in two seasons (2012 and 2013) and two populations (ASNA and ASNJ) (error bars signify standard deviation).

**Figure 3.**
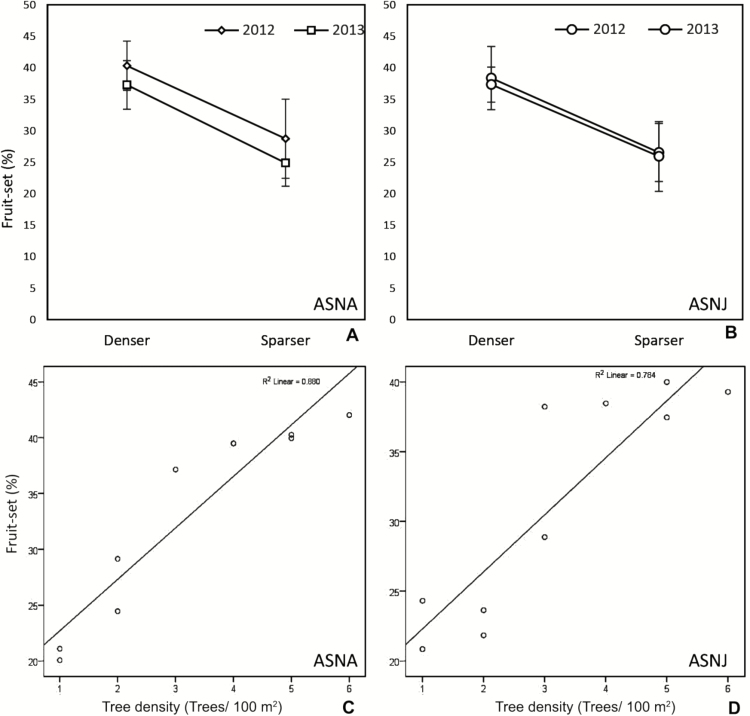
Effect of tree density on reproductive fitness. (A, B) Difference in mean percentage fruit-set in the denser and sparser plots in the two populations. In (C) and (D) the graphs represent correlation between the tree density (trees per 100 m^2^) and percentage fruit-set in the two populations.

The fly pollinator covered a significantly greater number of trees in a bout in the denser plots (3.2 ± 0.17, ±SE; pooled for 2 years and two sites) than those in the sparser (1.21 ± 0.09, pooled data) plots (Table 4). Moreover, the number of inflorescence probed by the flies in denser plot (10.18 ± 0.45, pooled data) was greater than the sparser ones (8.73 ± 0.4, pooled data) at both the sites (Table 4; Fig. 2A–D). Natural fruit-set (open-pollination) in denser plot (38.44 ± 0.46 %) was significantly higher than the sparser (26.52 ± 0.67 %) plot (*F*_1, 119_ = 229.573, *P* = 0.001; Fig. 3A and B). Moreover, fruit-set pattern strongly correlated with the density of trees per 100 m^2^ at ASNA (*R*^2^ linear = 0.88, Pearson correlation = 0.94) as well as at ASNJ (*R*^2^ linear = 0.78, Pearson correlation = 0.885) (Fig. 3C and D).

**Table 4.** Test between subject effect of three-way ANOVA for the pollinator behavior in denser and sparser plots. Here the number of inflorescence (glomeruli) visited per tree and tree visited per bout are considered as dependent variables while two populations, two seasons and two plots (denser and sparser) are considered as fixed factors. *P*-value = 0.05 > * > 0.01; ** < 0.01.

Dependent variable	Glomeruli visited per tree (df = 1,392)		Trees visited per bout (df = 1,192)	
Fixed factors	*F*	*P*	*F*	*P*
Population (ASNJ and ASNA)	21.93	0.000**	0.26	0.608
Year (2012–13)	0.50	0.47	0.09	0.758
Plot (denser and sparser)	9.37	0.002*	417.21	0.000**

## Discussion

The study shows that *A. sericea* var. *nummularia* is a partially self-compatible (PSC) tree species. However, selfing is not favoured and reproductive success in the species is majorly contributed through outcrossing. Dichogamy at the inflorescence level offers opportunity for success in outcrossing provided that its specialist pollinators (flies) are able to bring about pollination by covering unrelated trees in their foraging bout. Although genetic relationship among the trees in a foraging plot was not ascertained, significantly lower amount of seed weight from selfing than that from outcrossing indicated the prevalence of strong ID.

### Floral biology and pollination

*Anogeissus sericea* var. *nummularia* shows a specialized interaction with pollinator flies. Flies represent an important component of pollination guild in the arid and semi-arid environments (Ndoye *et al.* 2004; Chacoff *et al.* 2012) and are known to be pollinators of certain threatened plant species (Wiesenborn 2003; Murugan *et al.* 2006; Zych *et al.* 2014). Although the flowers appeared to be of generalist type, only the flies were recorded to visit the flowers. Considering that the flowering-handling time of the flies was prolonged (2–5 min), it is likely that such behaviour not only improved the pollen carry-over but also increased the chance of fruit-set by ensuring targeted pollen deposition on restricted surface of stigma. Pollen deposition is known to be facilitated by extended foraging on brush-blossoms, ensuring sufficient pollen deposition on to the stigma of most of the flowers within an inflorescence (Raju *et al.* 2012; Woodcock *et al.* 2014).

### Effect of density on pollinator behaviour and fruit-set

The pollinator frequency corresponded with the floral concentration in the trees. Increased visitation by the flies in denser plot can be attributed to their shorter flight distance and also to the amplified display of floral traits (Larson *et al.* 2001; de Lima Nadia and Machado 2014; Li *et al.* 2014). In aggregations, a mild fetid olfaction was apparent which was hardly perceivable around the sparsely located trees. Larger cumulative floral display in denser plot reduces the distance of successive visits by pollinators, and facilitates inter-plant pollinator movement (Pyke 1979). Flies being the territorial pollinators in general tend to visit denser patches to have maximum resources. Our study shows that there is significant effect of tree density on the number of conspecifics visited by pollinator in a bout. This is consistent with other studies associated with the density of conspecifics where pollinators are territorial (van Treuren *et al.* 1993; Franceschinelli and Bawa 2000). Flies tend to cover a greater number of trees in denser plot and confined their visit to a single tree in sparser plot.

It has also been argued that plant species with specialist pollinators are likely to compete for resource availability (Johnson *et al.* 2011). The increased floral density prompts increased pollinator competition among the trees that may decrease or have a neutral effect on net amount of fruit-set in the denser patches. However, in *A. sericea* the density appears to play a crucial role in facilitating cross-pollination rather than competing for pollination. Higher tree density is known to increase the combined display effect and attract a substantial number of pollinators with increased chances of pollen deposition (Sih and Baltus 1987; Kato and Hiura 1999).

Preferably, it would be rewarding for the pollinators to visit more flowers within a plant (optimal foraging) in sparser locations before moving to distant plants (Field *et al.* 2005). Some studies also implicate that specialized pollinators are not affected by the conspecific density (Aizen *et al.* 2002). Our findings on the foraging behaviour in sparsely located trees are also not in agreement with the ideal situation, as the flies visited a greater number of the glomeruli per tree in the denser plot. This deviation could be either due to the enhanced olfactory cues through aggregation of floral resources in the denser patches (facilitation) or competition-induced aggressive foraging (Hegland 2014).

The increased inter-tree movement among the conspecifics has a significant positive effect on the fruit-set in the species by enhancing the chance of cross-pollen deposition. Outcrossing floral contrivances such as protogyny in the flowers in the species reduces the chances of self-pollen deposition during intra-tree visit. However, as the pollination requirements of geitonogamy are similar to xenogamy, the slower movement of flies can also bring geitonogamous pollen load.

The proportional effect of plant density on fruit-set, and outcrossing, follows a common pattern across plant species (Ghazoul 2005). In *A. sericea*, the effect of tree density on the foraging pattern of pollinators and fruit-set was apparent. In both the populations, distribution pattern of trees became a limiting factor in reproductive success. Fitness attributes significantly correlated with the tree density within the populations. Lower fruit-set in isolated and sparsely distributed trees appears to be largely due to geitonogamy which is the primary cause of ID. The fruit-set pattern and the stigmatic pollen load in two plots also conform to the effects encountered through ‘Allee effect’ (Allee *et al.* 1949; Ghazoul *et al.* 1998).

Pollinator behaviour in a fragmented population can also be influenced through an edge effect, as microclimatic conditions may vary across the population and confine the pollinator more to the interior of the population (Jules and Rathcke 1999). Such consequences may appear similar to density-dependent effects on fruit-set as seen in the present study. However, our study does not clearly uncouple the effect of edge from that of density-dependent, possibly due to the involvement of a solitary specialized pollinator and its sheer attraction to the concentrated resources in the denser patch.

### Mating system

The degree of dependence on pollinators is believed to be directly associated with the breeding system of the plant species (Richards 1997). Furthermore, floral display size and outbreeding devices, especially dichogamy and herkogamy, can provide a balance between selfing and outcrossing within a population (Barrett and Eckert 1990; Harder and Barrett 1996; Herlihy and Eckert 2005). *Anogeissus sericea* is a partially self-compatible species with ISI to be around 0.41. The greater increased fruit-set in cross-pollinated flowers than self-pollinated ones suggests the prevalence of ID. In general, ID is stronger at early stages in predominantly outcrossed species while in self-compatible species there is manifestation of mild inbreeding at the later life-history stages (Husband and Schemske 1996). It has been argued that cross-pollination is favoured in the species when ID exceeds 0.5. In the present study, ID at different stages of life-history stages shows a very high value which is pivotal in the maintenance of outbreeding in the species (Table 2). The results also suggest that the fruit-set in denser and sparser plots is dependent on combinatorial factors which include floral trait and breeding system of the species. In denser plot within a population, pollinator visits on the protogynous flowers maximizes cross-pollination, thereby delimiting the detrimental effects of inbreeding on fruit-set.

## Conclusion

Our finding that there is protogyny in a biotically pollinated species is a deviation from the usual correlation seen between protogyny and evolution of abiotic pollination mode (Sargent and Otto 2004). Further, the tree species insufficiently fulfils the correlated evolution typically seen between protogyny and self-incompatibility (Routley *et al.* 2004). Tenets on the prerequisite of success of transition from self-incompatibility to selfing-compatibility argue that negative consequences on fitness of the progeny, arising from strongly deleterious alleles, must be purged. Otherwise consistent manifestation of ID at various life-history stages would prevent complete evolution of selfing (Busch 2005). *Anogeissus sericea* is a partially self-incompatible system, and selfing does not seem to be advantageous in the species. Many traits of the juvenile phase in the tree species are affected and do not seem to have purged to favour complete selfing. It is likely that the occurrence of protogynous dichogamy and high ID would continue to sustain better performance of offspring through outbreeding in the species. According to population genetic model, it is believed that outcrossing should prevail in populations when the value of ID exceeds 0.5 (Lande and Schemske 1985; Charlesworth and Charlesworth 1987; Dudash 1990). However, poor density of heterospecifics within the pollen carry-over range of flies appears to be an impediment in ensuring sufficient outcrossing in the species. As the tree species is threatened, reintroduction of unrelated sibs would be a crucial requirement along with the density of plantation.

## Sources of Funding

The work was supported by the Ministry of Environment, Forest and Climate Change, Government of India (22/9/2010-RE).

## Contributions by the Authors

V.K.S., C.B. and D.M. made equal contributions in conducting the research. R.T. and V.K.S. were involved in planning the research and in writing the manuscript.

## Conflict of Interest

None declared.
